# A Review of the Applications, Environmental Release, and Remediation Technologies of Per- and Polyfluoroalkyl Substances

**DOI:** 10.3390/ijerph17218117

**Published:** 2020-11-03

**Authors:** Jay N. Meegoda, Jitendra A. Kewalramani, Brian Li, Richard W. Marsh

**Affiliations:** 1 Department of Civil and Environmental Engineering, New Jersey Institute of Technology, Newark, NJ 07102, USA; jak93@njit.edu; 2 Princeton University, Princeton, NJ 08544, USA; lskbr02@yahoo.com; 3 Department of Chemical and Material Engineering, New Jersey Institute of Technology, Newark, NJ 07102, USA; rwm26@njit.edu

**Keywords:** PFAS, perfluorinated chemicals, remediation, organic pollutants, persistent pollutants

## Abstract

Per- and polyfluoroalkyl substances (PFAS) are pollutants that have demonstrated a high level of environmental persistence and are very difficult to remediate. As the body of literature on their environmental effects has increased, so has regulatory and research scrutiny. The widespread usage of PFAS in industrial applications and consumer products, complicated by their environmental release, mobility, fate, and transport, have resulted in multiple exposure routes for humans. Furthermore, low screening levels and stringent regulatory standards that vary by state introduce considerable uncertainty and potential costs in the environmental management of PFAS. The recalcitrant nature of PFAS render their removal difficult, but existing and emerging technologies can be leveraged to destroy or sequester PFAS in a variety of environmental matrices. Additionally, new research on PFAS remediation technologies has emerged to address the efficiency, costs, and other shortcomings of existing remediation methods. Further research on the impact of field parameters such as secondary water quality effects, the presence of co-contaminants and emerging PFAS, reaction mechanisms, defluorination yields, and the decomposition products of treatment technologies is needed to fully evaluate these emerging technologies, and industry attention should focus on treatment train approaches to improve efficiency and reduce the cost of treatment.

## 1. Introduction

Per- and polyfluoroalkyl substances (PFAS) are a large family of man-made fluorinated organic compounds whose widespread use and recalcitrant nature have led to their frequent detection in the environment, causing growing concerns over their impact on human health [1,2,3]. The origins of PFAS date to DuPont’s chemical research in the 1930s on polytetrafluoroethylene (PTFE), which was discovered unintentionally during research on stable fluorinated refrigerants [4]. Further research and development efforts led to the development of Teflon (the trade name of PTFE) in 1947, the most well-known of PFAS and one that remains in production still today, unlike many of its cousins [4]. Since then, the family has expanded into a diverse array of chemicals, with more than 4730 compounds having been identified [5].

The uses of PFAS are versatile and diverse. Its many formulations have seen use in applications such as firefighting foams/fireproofing, repellant cookware coatings, waterproofing, and surfactants, among others [6,7,8,9,10]. The marked commercial success and widespread use of PFAS can be attributed to its physical and chemical properties, such as a high degree of thermal and chemical stability due to the strength of its carbon-fluorine (C-F) bonds and its ability to reduce surface tension [10,11,12,13]. This is a double-edged sword; these very same properties cause PFAS to be bio-accumulative, environmentally persistent, and resistant to many current water treatment methods, leading these compounds to be nicknamed “forever chemicals” [14,15,16]. Global studies have identified significant concentrations of PFAS at both urban and rural sites on all six continents, with many sites located at far distances from potential sources, such as in the Antarctic [17,18,19,20,21,22]. Throughout these sites, PFAS have been detected in air, water, soil, plants, and animal tissues [23,24,25]. The presence of PFAS in animal tissues and food packaging has been cited as an important pathway for human exposure to PFAS, along with other forms of environmental exposure [26,27].

This paper aims to provide a broad overview of PFAS in the environment as well as its remediation through different technologies. It begins with a discussion of the health effects of PFAS and the limits put in place by U.S. federal and state agencies. Then, the properties of PFAS are examined to provide context for a discussion of its environmental release, fate, and transport. Finally, various existing and emerging treatment technologies and approaches are described with their advantages and disadvantages. This review was performed using a methodical search of PubMed, Scopus, and the Web of Science databases. In doing so, 295 relevant publications were identified after an initial exclusion. The authors then selected a limited list that was supplemented by government publications and gray literature.

## 2. Health Concerns and Regulations

**Figure 1:**
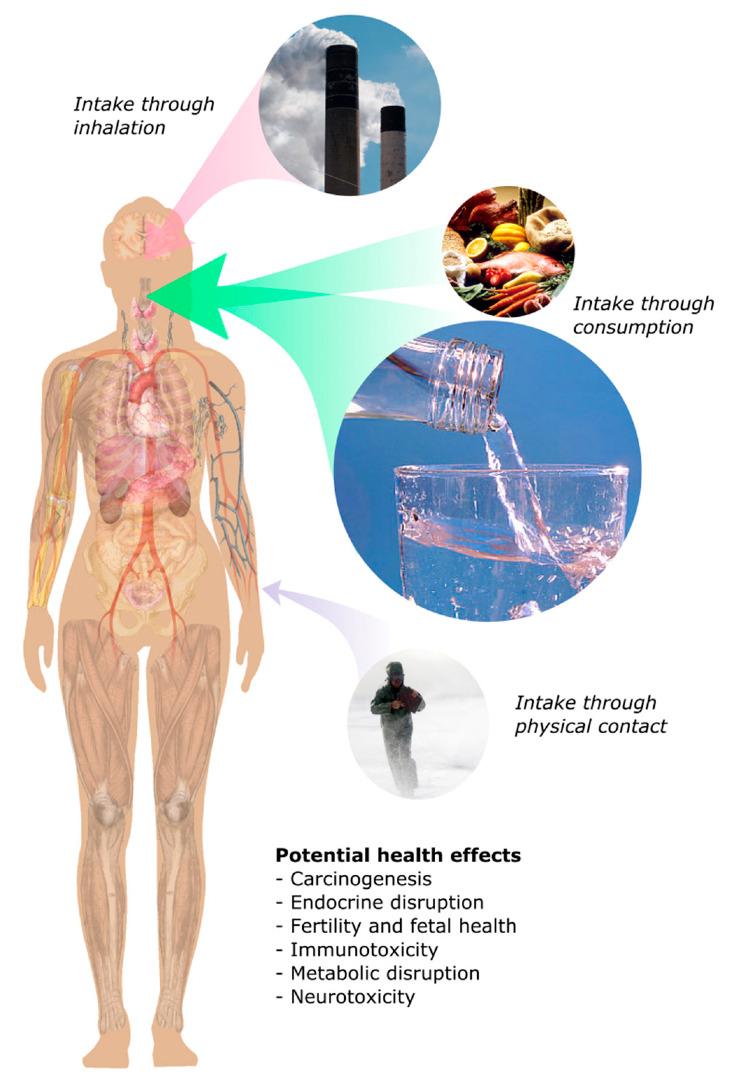
Pathways of direct human per- and polyfluoroalkyl substance (PFAS) exposure and its potential health effects [31,32,33,35,36,37,38].

Research into the health effects of PFAS has led to increased concern and governmental regulation, and long-term studies have revealed its potentially harmful health effects [28,29]. Additionally, several large, ongoing biomonitoring studies are currently underway, most notably the National Health and Nutrition Examination Survey conducted by the United States Centers for Disease Control and Prevention [30]. Several decades of research have led to numerous findings, including evidence of PFAS in the blood serum of chemical workers, a finding from the 1980s [31]. PFAS are water-soluble, which allows them to behave differently from many other organic pollutants when interacting with the human body. For example, they have a strong tendency to interact with tissue and serum proteins such as albumin, which may contribute to their presence in livers, kidneys, and blood [32,33]. Zebrafish studies have confirmed their toxic and neurotoxic effects, with increased neurotoxicity correlated with increased carbon chain length [34]. Longitudinal studies in humans have discovered that PFAS lead to altered kidney and thyroid function [35]. A variety of epidemiological studies conducted in recent years have identified possible effects including, but not limited to, endocrine disruption, carcinogenesis, immunotoxicity, and metabolic disruption [36,37,38]. These effects are depicted in Figure 1 where the size of the arrow qualitatively depicts relative PFAS intake amounts. Due to its high solubility, most PFAS enters the human body through drinking water.

Several major manufacturers have also joined a voluntary perfluorooctanoic acid (PFOA) phase-out program launched by the USEPA in 2006 [39,40,41]. Further regulatory steps have included the adoption of provisional health advisory values in 2009, the inclusion of six PFAS in the Third Unregulated Contaminant Monitoring Rule (UCMR) for required monitoring in 2012, and lifetime health advisories for PFOA and perfluorooctanesulfonic acid (PFOS) in 2016, with the latter stating that exposure to the combined concentration of PFOA and PFOS over 70 ppt may result in adverse health effects [41,42,43,44].

Unlike many other legacy contaminants (e.g., polychlorinated biphenyls (PCBs), lead), the regulation of PFAS poses several unique challenges. PFAS are among the first compounds to be screened and regulated at very low concentrations (in the parts per trillion (ppt) range) [45]. In comparison, contaminants such as benzene and trichloroethylene are regulated in part per million (ppm) or part per billion (ppb) range. PFAS may be released into the environment by a variety of means: (1) industrial point sources and their resulting emissions re-entering surface water and groundwater, (2) spillages and runoff from incidents and locations utilizing firefighting foams, (3) landfills, and (4) wastewater treatment plants and the application of contaminated biosolids. This is further complicated by the hydrophobic, lipophobic, and surfactant properties of PFAS, which, when combined, confer unique fate and transport characteristics [46]. PFAS are miscible, so they do not exist as a separate non-aqueous phase, unlike some hydrocarbons and solvents. The persistence and mobility of PFAS in the environment make it a serious long-term concern. As a result of growing recognition of its persistence, the rate at which guidance and regulations are being developed by state and federal authorities has rapidly increased over recent years, resulting in some of the regulations listed above [9,47,48]. Regulatory agencies are also beginning to expand their regulation of PFAS to cover additional emerging PFAS and to extend regulations beyond drinking water and groundwater, resulting in increased overall regulatory scrutiny.

As the small list of regulated PFAS slowly grows, some PFAS precursors have also begun to be subjected to research scrutiny [11,49,50]. Currently, many U.S. state regulatory agencies (e.g., California, Michigan, New Hampshire, New Jersey, New York, and Vermont) require the monitoring of an expanded list of PFAS through the inclusion of precursors beyond federal requirements or having more stringent standards [51,52]. Currently, regulatory guidance and research attention exists for PFOS, PFOA, and a variety of other PFAS, including perfluorononanoic acid (PFNA), perfluorobutanoic acid (PFBA), pentafluorobutanesulfonic acid (PFBS), perfluorohexanesulfonic acid (PFHxS), perfluorohexanoic acid (PFHxA), perfluoropentanoic acid (PFPeA), perfluoroheptanoic acid (PFHpA), perfluorooctanesulfonamide (PFOSA), and perfluorodecanoic acid (PFDA) [51].

Emerging PFAS, the “shorter-chain” alternatives (e.g., GenX, PFBS) to legacy PFAS, have been developed to complement and/or phase-out legacy PFAS, a change brought about by increased regulatory pressures. Most legacy PFAS are long-chain compounds with proven bio-accumulative properties, whereas most emerging, shorter-chain PFAS have been promoted by the chemical industry as safer and more environmentally sustainable alternatives that should reduce the potential for accumulation in humans [53]. However, recent research scrutiny has found that both long-chain and short-chain PFAS are still persistent pollutants that require remediation [54,55,56]. This is complicated by gaps in knowledge about emerging PFAS and their potential bio-accumulative and toxic effects [57,58]. These difficulties have led some experts to question whether emerging/replacement PFAS are indeed a safer alternative to legacy PFAS [59].

## 3. Classification of PFAS

**Figure 2:**
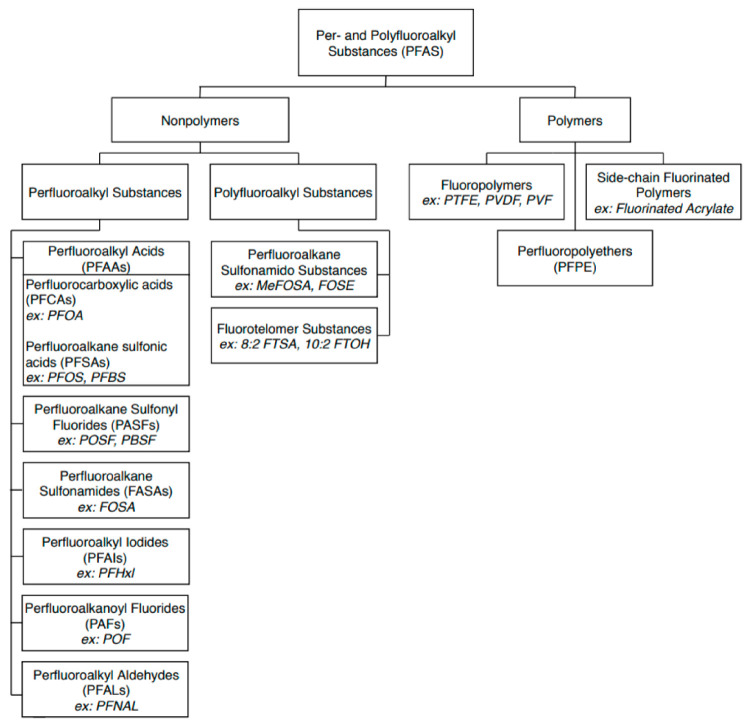
Classification tree of PFAS family compounds adapted from Buck et al. [3] and ITRC PFAS Team [10].

PFAS can be broadly divided into two main classes: polymers and non-polymers. While the polymer class contains commonly used substances such as PTFE (Teflon) and ethylene tetrafluoroethylene (ETFE, trade name Tefzel), most research into PFAS focuses on non-polymers, as they are more widely detected in the environment and thus are more likely to be the subject of government guidelines and regulations [3,10]. The non-polymer class is further divided into two subclasses: perfluoroalkyl and polyfluoroalkyl substances. Perfluoroalkyl substances contain fully fluorinated carbon chains where all possible bonding sites on the carbon atoms have been replaced by fluorine, except for one site on the terminal carbon, where a functional group is attached. Polyfluoroalkyl substances are only partially fluorinated, containing a non-fluorine atom (typically hydrogen or oxygen) bonded to at least one, but not all, carbon atoms, while at least two or more of the remaining carbon atoms in the carbon chain tail are fully fluorinated. Within the perfluoroalkyls is yet another subdivision: perfluoroalkyl acids (PFAAs). This group contains some of the most frequently detected PFAS, including perfluorooctanoic acid (PFOA) and perfluorooctane sulfonate (PFOS). Two important subgroups of PFAAs are the perfluorocarboxylic acids (PFCAs) and perfluoroalkane sulfonic acids (PFSAs), each distinguished by their respective carboxyl and sulfonate functional groups. An in-depth PFAS family tree is illustrated in Figure 2.

## 4. Physical and Chemical Properties

A thorough comprehension of the physical and chemical properties of PFAS is vital for understanding their release into the environment and the difficulties related to their treatment [60]. These unique properties are primarily associated with the fluorine atoms that replace hydrogen along PFAS carbon chains. The high electronegativity and small size of fluorine make the C-F bond one of the strongest covalent bonds in nature. The large amount of energy required to break this bond is responsible for the stability of PFAS in the presence of oxidants and high temperatures as well as its resistance to chemical and biological degradation [13,60]. Further substitution of a carbon atom only increases the bond strength due to the shortening of the bond length, as the carbon center becomes increasingly positive [61]. Additionally, the low polarizability of fluorine imparts PFAS’s signature property of simultaneous hydrophobicity and lipophobicity [62].

While the C-F bond is common to all PFAS, there is significant structural variation across these compounds. Carbon-chain length is often used in the classification of PFAS, as it has a strong impact on a compound’s properties. Increasing chain length is associated with increasing lipophilicity and hydrophobicity [60,62]. PFAS can be found in both aqueous and solid matrices, although longer-chain PFAS are more likely to attach to soil particles due to differing adsorption potentials. These differences in aqueous solubility and adsorption potentials cause disparities in mobility and environmental transport [29]. Therefore, the distribution of long-chain and short-chain PFAS in the environment is highly dependent on chain length. While important, chain length is not the only determinant for properties of PFAS.

Functional groups, such as carboxyls, sulfonates, and hydroxyls, also affect the chemical properties of PFAS. For example, more electrophilic PFCAs, which contain carboxyls, are easier to degrade than PFSAs, which contain sulfonates, when they have the same number of carbons [63]. Functional groups can also dissociate into the ionic form in aqueous solutions under appropriate conditions. Depending on the functional group, the ion formed can either be an anion, a cation, a zwitterion, with anions being more prevalent in the environment [64]. Different ionic forms behave differently in the environment [65]. Cations are more likely to be adsorbed to soils, which typically have a net negative charge, whereas the sorption of anions is generally lower [66]. pH can further complicate the environmental transport of ionic forms. For instance, under higher pH values, the electrostatic repulsion from deprotonated oxides can suppress anion sorption onto soil surfaces [67].

### PFOA and PFOS

As the most widely produced PFAS in the United States, PFOA and PFOS are widely detected in the environment [68]. Thus, they offer a very clear example into the effect of physical and chemical properties of PFAS on their environmental fate. As PFAAs, both contain fully fluorinated hydrophobic tails and a hydrophilic functional group. PFOA and PFOS are mostly inert, water-soluble acids that exist predominantly in the anionic form along a wide range of pH values. Between these two compounds, PFOA is found mostly in the dissolved phase, while PFOS has a higher sorption capacity [69]. PFOS has also been shown to exhibit a greater tendency to bioaccumulate due to its longer perfluoroalkyl chain length [68,70]. The biotic and abiotic degradation of most polyfluoro-substances (i.e., non-fully fluorinated PFAS) results in the formation of PFAAs [25,71]. These polyfluoroalkyl substances that degrade to create terminal PFAAs are referred to as “precursors.”

Additional physical and chemical properties comparing PFOA and PFOS, in addition to several other PFAAs, are listed in Table 1. These values will provide context for a discussion on the fate and transport as well as the remediation of PFAS.

## 5. Environmental Release

**Figure 3:**
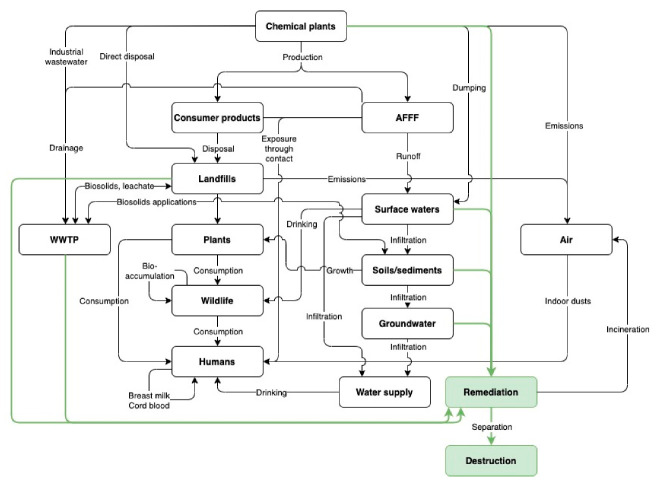
Pathways of PFAS environmental release and exposure [6,10,77,78,79,80,81,82,83,84,85,86,87,88,89,90,91,92,93,94].

The widespread use of PFAS has greatly contributed to their presence in the environment. They have been found in a variety of environmental matrices including surface waters, groundwaters, wastewaters, solid wastes, soils, and sediments [72,73,74,75,76]. Although the extent and sources of release are wide and diverse, major PFAS point sources in the United States and abroad can be largely divided into four major categories: (1) industrial facilities, (2) areas utilizing Class B firefighting foams, (3) solid waste management facilities, and (4) wastewater treatment facilities and their associated discharge areas. Figure 3 illustrates these potential releases, absorption pathways, and paths to their final destruction.

### 5.1. Industrial Facilities

Facilities involved in the production or utilization of PFAS have been associated with their environmental release, with variable release mechanisms and released compounds. The 3M Corporation was a major producer of select PFAS through the 20th century, and significant groundwater and well contamination have been found within the vicinity of their disposal facilities [95,96]. Studies have found elevated PFAS levels in the blood serum and incidences of cancer in people who reside within the vicinity of these facilities [87,88]. Before extensive regulation, facilities were regularly discharging wastes into surface waters and the air, with atmospheric transport being a key factor in extending the range of pollution to up to 30 miles [87,97,98,99]. This has raised an acute need for extensive biomonitoring in areas near PFAS-producing facilities.

### 5.2. Firefighting Foams

Class B firefighting foams, particularly aqueous film-forming foams (AFFF) designed for extinguishing flammable liquid hydrocarbon fires, frequently utilize PFAS due to their unique properties [89]. Class B fluorine-containing firefighting foams are commercial surfactant solutions that have been (and continue to be) stored and used for fire suppression and training. These foams have seen extensive use in areas with high risks of fires, most notably on airfields and military installations [90,100,101,102,103]. Many of these facilities were not designed with AFFF containment as a priority [100]. Consequently, these design oversights can allow PFAS to enter into surrounding soils and waters through leaching and run-off, often displaying leaching at significant concentrations [6,89,91,92,93]. Around AFFF pollution sources, PFAS in groundwater and surface waters can exceed USEPA drinking water advisories by three to four orders of magnitude [102]. At these concentrations, PFAS present in surface waters can easily accumulate in aquatic life and sediments [90,94].

### 5.3. Solid Waste Management Facilities

PFAS are used in many consumer products, such as clothing fabrics and food wrappings [104]. At the end of their useful lives, many of these products are landfilled. Apart from consumer goods, other sources of PFAS in landfill leachate include contaminated industrial waste, sewer sludge, and waste from contaminated sites [10,105,106]. The presence of PFAS, including PFAAs, fluorotelomer polymers (FTPs), perfluoroalkyl sulfonamide derivatives, and polyfluoroalkyl phosphate esters (PAPs) in landfill leachate is well documented, though the range of concentrations varies widely [104,107,108]. In particular, PFAAs such as PFCAs and PFSAs are the most frequently detected PFAS in landfill leachate in the ng/L to mg/L range [109,110]. Furthermore, short-chain PFAAs (four to seven carbons) are more commonly found in landfill leachates when compared to long-chain PFAAs (more than seven-carbons) [77,110]. In the United States, PFCAs and PFSAs concentrations in landfill leachate were reported to range from 10–8900 ng/L and 50–2300 ng/L, respectively [104,111,112]. Although leachate treatment is designed to remediate potential groundwater contaminants, biological leachate treatments have been found to increase PFAS concentrations as opposed to non-biological treatments, possibly through the formation of additional PFAS through the biodegradation of precursors [106,113]. This fits in line with the general trend of bioreactor landfills having higher PFAS concentrations than non-bioreactor landfills [112]. Atmospheric transport has also garnered recent attention due to the semi-volatility of some PFAS precursors and their presence in ambient landfill air, creating a potential need to investigate landfill gas emissions [109,114,115]. Solid waste landfills allow for the environmental release of PFAS laden leachate through discharge to wastewater treatment facilities, infiltration to groundwater from unlined landfills, accidental release due to leakage from landfill liners, and volatile gas emissions through the top cover [10].

### 5.4. Wastewater Treatment Plants

Wastewater treatment plants (WWTPs) receive PFAS through influents, including discharge from consumer and industrial point sources, landfill leachates, and firefighting foam runoff [116]. WWTPs have been found to be a significant point source for PFAS release, often because WWTPs are not designed to remediate persistent organic pollutants [78,79]. Wastewater can have up to 1000-times greater PFAS concentrations than the surrounding receiving waters, and although receiving water bodies can dilute the PFAS concentration, it often fails to dilute it to a concentration below drinking water advisories [78,117]. Several studies also report elevated PFAS concentrations in effluents due to precursor degradation [78,117,118,119]. After wastewater treatment, much of the biosolids produced are diverted for reuse in land-based applications, providing another avenue for release. In the United States alone, annual PFAS loads from biosolids are between 2749–3450 kg/year, which is a sufficient to create strong contamination concerns [80,81]. This is particularly concerning, given the increased use of biosolids in recent years and the constant recirculation of PFAS between WWTPs and landfills [119]. WWTPs receive PFAS-laden leachates, and WWTP biosolids are often deposited back in landfills, creating a cycle [77,82,83]. Thus, through point source discharges of effluents, leakage or unintended releases from surface impoundments and structures, air emissions, biosolid disposal and management, and precursor reactions during treatment, WWTPs can become major contributors to the environmental release of PFAS.

## 6. Fate and Transport

Due to the diverse physical and chemical properties of PFAS as outlined above, their behavior in the environment is highly complex. Variations in hydrophobicity and lipophobicity across the PFAS family unlock a wide range of possible transport pathways and are a cause of their widespread distribution in the environment. The movement of PFAS in the environment primarily occurs through atmospheric transport, aqueous transport, abiotic solid-phase partitioning, and bioaccumulation in plants, humans, and wildlife. A representation of these pathways beginning with the production of PFAS at a chemical plant, is illustrated in Figure 3.

### 6.1. Atmospheric Transport

While generally less volatile than other organic contaminants, PFAS can partition to the atmosphere under certain conditions. Neutral PFAS such as fluorotelomer alcohols (FTOH), fluorotelomer olefins (FTO), fluorotelomer acrylates (FTAC), and fluorosulfonamido alcohols (FSA) are considered to be volatile or semi-volatile compounds and have been detected in the atmosphere in the gaseous phase [21]. The presence of PFAS in remote areas such as in the Arctic with concentrations as high as 26 pg/m^3^ for 8:2 FTOH can likely be attributed to the atmospheric transport of these volatile compounds [71,120] It has been hypothesized that the simultaneous presence of PFOS and the absence of PFCAs in the Antarctic indicate that the occurrence of PFOS in remote areas is a result of the photochemical oxidation of volatile precursors rather than aqueous transport [20]. Due to their low volatility, ionic PFAS can be transported in the atmosphere while attached to particulate matter, and several studies have detected the presence of PFSAs and PFCAs in this phase in the environment [121,122,123].

### 6.2. Aqueous Transport

The high solubility and persistence of some PFAS in water, particularly PFAAs, contribute to their frequent detection in the aqueous environment and considerable potential for long-range aquatic transport [71,124]. Differences in hydrophobicity due to chain length impact the mobility of PFAS in water. Short-chain molecules have a lower hydrophobicity and higher solubility in water (see Table 1), resulting in preferential partitioning to the aqueous phase compared to long-chain molecules [125,126,127]. Due to the competing tendencies of the hydrophilic head and lipophilic tail that give PFAS their low surface tension, PFAS tend to accumulate at air–water interfaces. This observation provides useful information for selecting PFAS remediation methods [128]. A further consequence of its low surface tension is the formation of micelles at higher concentrations. The critical micelle concentration (CMC) for PFOS and PFOA has been cited as 3500 mg/L and 5800 mg/L, respectively [129]. It has been hypothesized that the formation of micelles could enhance adsorption onto carbon and other minerals while blocking diffusion into the inner surface of the adsorbent [130]. While the CMC is unlikely to be reached in the environment, it may play a role in the behavior of PFAS in highly concentrated waste streams, such as those which result from effluents of ion exchange resins and reverse osmosis. Vo et al. 2020 and Rahman et al. 2014 provide comprehensive reviews of PFAS in the aqueous phase, wherein Vo et al. 2020 investigated the behavior and remediation of PFAS in water and wastewater while Rahman et al. 2014 looked specifically into PFAS in drinking water [131,132].

### 6.3. Abiotic Soil Partitioning

There are two processes by which PFAS partition to solid-phase materials: (1) electrostatic interactions between clay particles and (2) polar head group and hydrophobic interactions between organic soil matter and carbon tails [69,133]. For electrostatic interactions, oxides present in soils and sediments provide positively charged surfaces that attract the negatively charged functional heads of anionic PFAS [130]. When soil surfaces are negatively charged, divalent cations can act as bridges, effectively shifting negatively charged sites into positively charged sites [130,134,135]. Different site characteristics can greatly influence sorption due to different levels and types of electrostatic interactions. Changes in pH can also alter surface charges such that the sorption of anionic PFAS increases with decreasing pH [69]. The content and properties of organic matter present in soils and sediments play an important role in the hydrophobic interactions of C-F chains in PFAS. Studies have demonstrated that the sorption of PFAS onto negatively charged surfaces is possible, despite the electrostatic repulsion, with higher sorption amounts for more hydrophobic compounds [130]. The simultaneous hydrophobicity and oleophobicity of the tail create competing tendencies, impacting sorption onto adsorbent surfaces that are both hydrophobic and oleophilic. Therefore, it is likely that both hydrophobic and electrostatic interactions affect the partitioning of PFAS to the solid phase [69]. A detailed review of the characteristics and treatment of PFAS in soil can be found in the investigation by Mahinroosta and Senevirathna, 2020 and Li et al. 2018 [74,133].

### 6.4. Bioaccumulation in Organisms

The bioaccumulation of PFAS allows for transport between organisms. As shown in Figure 3, PFAS present in the environment can be transported to plants and other wildlife. In plants, PFAS released from landfills and WWTPs are deposited in soils and up-taken via diffusion into plants’ root systems [84,85]. Crop research indicates that uptake and storage primarily occur in the vegetative compartments but are also highly dependent on plant species [136]. From plants, PFAS travel up the food chain through consumption, biomagnifying in organisms at the top, typically carnivorous predators. For example, a study in the state of Washington found PFAS concentrations of up to 910 ng/g in osprey eggs, while concentrations in fish liver tissue did not exceed 530 ng/g [137]. PFAS binds to serum albumin and fatty acid-binding proteins in the human body, unlike more lipophilic contaminants such as PCBs, DDT, and mercury [124,138]. With albumin being a major protein in mammalian milk, breastfeeding is an additional transport route between organisms [33,139]. Especially in humans, the ingestion of PFAS can occur from exposure to consumer products, consumption of contaminated food, and contaminated water and breastmilk drinking. The half-lives of PFAS in the human body have been found to be longer than any other mammal, with long-chain PFAAs lasting up to several years [86]. This persistence and widespread distribution throughout ecosystems highlight an urgent need for novel and efficient PFAS removal and remediation methods.

## 7. Treatment Technologies and Challenges

PFAS have become widely distributed in the environment, and they are detected in surface water and groundwater at hundreds of locations in the US and around the world [2,3,102,140,141,142]. Growing toxicology research on this contaminant class has led to increased societal and regulatory awareness [14,28,51]. Many treatment technologies aim to remediate PFAS by taking advantage of their physical and chemical properties. Many existing water treatment technologies (e.g., air sparging, enhanced aerobic bioremediation, etc.) have shown limited success in mineralizing PFAS, as PFAS are uniquely recalcitrant and persistent [143]. Advanced oxidation processes (AOPs), which use hydroxyl radicals such as Fenton’s reagent ($H_{2}O_{2}$ and ${\mathrm{Fe}}^{2+}$) and ozone ($O_{3}$), have shown limited effectiveness in the mineralization of PFAS due to the strength of the C-F bonds and the high electronegativity of fluorine [143,144,145]. Some transformation or destruction technologies, including advanced oxidation, biodegradation, advanced reduction, and thermal destruction, are currently being tested [146,147,148,149].

Treatment technologies for PFAS in environmental matrices are still evolving. The final objectives of remediation processes are defined by a plethora of guidelines and policies from all levels of government; these competing guidelines can complicate the development of remediation methods [28,51,146,150]. There is considerable uncertainty on the endpoints of PFAS remediation strategies, and because there are very few currently available technologies, the environmental industry likely needs to prioritize protecting drinking water sources and human health. Thereby PFAS remediation work has focused primarily PFAS-impacted liquid streams (e.g., groundwater, drinking water, landfill leachates, wastewater, and industrial discharges) that are directly or indirectly affecting the drinking water sources through novel sequestration and/or removal methods [28,146].

Sequestration remains the most common treatment for PFAS-contaminated liquids, although sequestration fails to destroy PFAS [151,152]. Sequestration process are considered as interim actions and involve the physical mass transfer (sequestration) of PFAS. These approaches process extremely large volumes of liquid with low PFAS concentrations, typically in part per trillion range (ppt) to low part per billion range (ppb) and yield a much smaller volume of concentrated PFAS, which requires subsequent disposal or incineration [153]. Generally, there is a concerning paucity of literature detailing the destruction mechanisms and final fates of PFAS under existing and developing treatment methods that must be addressed with further investigation [147,154].

### 7.1. Current Water Treatment Technologies

The most common and effective treatment processes for PFAS are filtration technologies (e.g., nanofiltration (NF), reverse osmosis (RO)) and adsorption processes (e.g., granular activated carbon (GAC) and ion exchange resins (IX)) [146,155,156].

Filtration can remove contaminants, including PFAS, by forcing pressurized contaminant streams through a semipermeable membrane [146,157,158]. A variety of membrane filtration methods have been tested for PFAS removal, and results have shown that RO and NF are effective in removing PFAS, while microfiltration (MF) and ultrafiltration (UF) perform poorly [16,151,159]. RO and NF have also shown good performance on PFAS of all chain lengths, and this performance could be reasonably expected to translate to PFAAs and their precursors [148]. At the same time, RO and NF have high implementation and operation costs, which might be prohibitive [148]. The membranes may also become compromised by suspended solids and the geochemistry of water, which can create additional costs due to fouling [160]. Other fundamental limitations, such as subpar efficiency, contaminant rejection, and low throughput flow rates, can constrain the range of useful applications [155].

Adsorption is a physical mass transfer process that binds molecules of PFAS to the surface of adsorbents through electrostatic and hydrophobic interactions [130,161]. GAC sorption, a hydrophobic process, is currently the most widely used treatment technology for PFAS-contaminated water [130,148,155,162]. GAC sorption effectively removes longer carbon chain PFAS with more C–F bonds and becomes progressively less effective for removing shorter carbon chain compounds and their precursors [163,164]. Surface area, macroscopic pore size, and surface chemistry are sorbent properties which have a great deal of influence over the PFAS uptake [164,165]. Thermal destruction at temperatures higher than 1000 °C can also be used for the reactivation of GAC surfaces [153,166,167].

Anion ion-exchange (IX) resins can effectively adsorb aqueous PFAS through ion exchange and adsorption [168]. IX resins consist of synthetic polymers with positive exchange sites, which ionically bond with negative sulfonic and carboxylic acid heads of PFAS in aqueous media under typical pH values [146,148,168,169]. The hydrophobic carbon-chain tail is able to bind to the hydrophobic backbone and cross-links on the resin surface. The result of these dual mechanisms allows for high adsorption, short contact time, and small equipment footprints that improve the efficiency of IX resins in comparison to other methods (e.g., GAC) which function strictly by adsorption [168,170,171,172]. A number of intake parameters of treated water, including concentration of co-contaminants, competing inorganic ions, or impurities, can foul or degrade performance of IX resins and may require pretreatment steps to optimize the performance of sorption technologies. IX resins can be categorized as either single-use or regenerable [146,148]. Single-use resins are used until a breakthrough occurs, after which they are incinerated or landfilled, while regenerable resins can be restored using a regenerant solution [168]. A brine solution which combines solvents such as methanol with sodium chloride or sodium hydroxide is used to generate ion-exchange membranes [168,173].

RO and other filtration processes are highly effective in removing PFAS of all chain lengths [148,155]. However, these filtration systems (particularly RO) are expensive when compared to GAC and IX, which may be why the latter options remain the most common. More importantly, IX can be used repeatedly, improving sustainability and reliability [168,173,174]. Currently, a limited number of full-scale regenerable IX systems have been installed by one commercial vendor, ECT2 [146]. However, this technology is gaining much commercial interest, and soon there will likely be several regenerable IX systems installed by multiple vendors across the US.

Remediation by adsorption constitutes an interim action, undertaken to comply with drinking water advisories. A concentrated secondary waste stream often results, which may have a solid phase, as in the case of GAC treatment, or a liquid phase, as in the case of regenerated IX resins and filtration [149,168,175]. Ultimately, these waste streams must undergo secondary handling and disposal, frequently through landfilling or incineration [28]. Ex-situ incineration is by far the most common method, although it has raised environmental and public health concerns [28,153]. In addition, incomplete PFAS mineralization and incineration produce by-products with long atmospheric half-lives (e.g., perfluoroisobutylene, fluorocarbons, and fluoroalkanes) or PFAS discharges into the environment [153,167,176].

### 7.2. Emerging Water Treatment Technologies for PFAS

Foam fractionation is an emerging sequestration technology [155,177]. The functional head group of PFAS is hydrophilic, but the tail is hydrophobic. This makes the air–water interface of a bubbles highly suitable for sequestering PFAS. Foam fractionation exploits the surfactant properties of PFAS by sparging air through a column with PFAS-contaminated liquids, creating a PFAS-enriched foam that can be easily removed [148]. Using a series of narrow columns, PFAS are progressively removed until compliance with drinking water regulations is reached. Foam fractionation is also being adapted with ozone, a patented process by Evocra, which replaces air with ozone [178,179]. In this process, fine ozone bubbles are used, which oxidize non-fluorinated organic compounds in water. This confers an advantage in that both organic contaminants and PFAS can be removed simultaneously. It has been further shown that ozone fractionation has a higher removal efficiency than air fractionation [180]. However, they too, generate concentrated waste streams requiring further processing, creating a 1000-fold concentrated effluent at 0.5–2% of the influent volume [148].

Many advanced redox reactions (e.g., electrochemical oxidation, photolysis, photocatalysis, aqueous reduction processes using aqueous iodide or dithionite and sulfites, high-voltage electric discharges, microbial treatment, ozonation under alkaline conditions, permanganate oxidation, sonolytic degradation, sub or supercritical treatment, and microwave-hydrothermal treatment) have shown varying degrees of effectiveness in degrading PFAAs in water [181]. Electrochemical oxidation, plasma, sonolysis, and hydrothermal/supercritical oxidation are some emerging technologies that are transitioning from lab-scale research to field-scale testing [2,28,148,153]. While promising, these technologies need extreme operating conditions, centralized laboratory equipment, and high chemical and energy requirements, impeding their practicality and widespread use [182]. However, optimizing PFAS remediation by combining concentration and eventual destruction in a treatment train can offer energy-efficient and cost-effective options for decision-makers [146,182].

Electrochemical treatment is being exploited to treat PFAS, and it is actively transitioning from laboratory-scale to field-scale demonstrations [183]. Electrochemical treatment uses anodic oxidation, a process of direct electron transfer from an anode to molecules within electrochemical cells equipped with anodes, cathodes, and electrolytes [184,185]. Electrochemical cells with mixed metal oxide anodes are effective for treating PFOA and PFOS when currents are applied at densities of 1–50 mA/cm^2^ [155]. Mixed-metal anodes, including lead dioxide, titanium oxide, tin oxide, and boron-doped diamond (BDD) have been tested as electrode materials, with BDD showing advantages due to commercial availability, high reactivity, low absorptivity, and flexibility [63,186,187]. Numerous operating parameters such as pH, current density, electrolyte type, electrode distance, PFAS concentration, and temperature all have a strong influence on the effectiveness of electrochemical oxidation. During a typical residence time of 4–10 h, a PFAS molecule undergoes anodic oxidation, which removes carbon atoms through decarboxylation pathways. This process repeats, continually shortening the molecule and eventually producing fluoride and sulfate ions.

In electrochemical treatment, the formation of toxic by-products (e.g., hydrogen fluoride, chlorine gas, bromate, perchlorate, and organic halides) is a recurring issue [63,155,188]. Heavy-metal electrodes, which are toxic, may also slowly break down and release into the environment due to wear [184]. In electrochemical oxidation, PFAS undergoes in sequential defluorination, which creates short-chain PFAAs [147,149]. This may become problematic, as electrochemical oxidation is not as effective in defluorinating short-chain PFAS, which may lengthen residence time [187,188].

Plasma treatment applies pulses of high-voltage electrical discharges through contaminated liquids with an electrode directly through the liquid or through bubbles to a ground electrode within the liquid [189,190]. These discharges react with water molecules and alter the physiochemistry of the liquid through powerful electric fields, intense UV radiation, and shock waves. These effects result in the formation of strong reactive species (e.g., hydrogen radicals (H•), oxygen radicals (O•), hydroxyl radicals (OH•), hydrogen peroxide (H_2_O_2_), aqueous electrons, H_2_, O_2_, and O_3_), aqueous electrons, and hot plasma electrons, which, in sum, are conducive to the treatment and removal of contaminants [189,191,192]. These highly reactive species react with anionic PFAAs, resulting in decarboxylation pathways which cleave C-C bonds. These species react with anionic forms of PFAAs, resulting into the decarboxylation degradation pathways of C-C bond cleavage, creating progressively shorter chains and various ions. As a result of the numerous moving parts of such a reaction, plasma technology depends on numerous technical parameters, including conductivity, electrode material, power, pulse repetition, and voltage, among various other physiochemical properties of the treated contaminant stream [147]. This process is highly energy-intensive, and its reaction efficiency can be compromised by geochemical components present in the liquid [153,193,194]. The reaction mechanism for plasma treatment is not well understood, and existing research suggests that there are multiple parallel destruction pathways which take place during treatment. Furthermore, while the degradation of PFOA and PFOS are relatively well-understood, the effectiveness of plasma treatment on short-chain PFAS is unclear. PFOS decomposition by DC plasma has also been demonstrated, albeit with PFOS showing reduced degradation due to its sulfur group when compared with PFOA and its carboxyl group [193]. Additionally, plasma-based water treatments have limited full-scale application for other contaminants, and its reactions with other by-products have yet to be fully elucidated [146].

High-frequency ultrasound has also been demonstrated to be effective and relatively fast in mineralizing PFAS, although existing research largely exists at the bench scale [143,145,195,196,197,198,199]. The sonolytic degradation of PFAS is believed to be caused by high-temperature pyrolysis at the surface of imploding cavitational bubbles [145,155,198]. PFAS degradation is believed to be enhanced by the presence of salts and higher initial concentrations, which are key attributes of concentrated waste streams [143,198]. Pyrolytic decomposition pathways are likely further applicable to all PFAS, destroying shorter-chain PFAAs and polyfluorinated precursors as well as more commonly studied PFAAs (e.g., PFOA and PFOS) [198]. Unlike methods that utilize sequential defluorination pathways, sonolysis can completely mineralize PFAS [143,145]. The scaling-up of sonolytic remediation processes presents a steep design challenge as a variety of parameters related to the design of a reactor (e.g., size, geometry, transducer locations, flow, etc.) can influence the distribution of cavitational events [195,200,201]. However, it is generally believed that flow-through reactors can decrease energy consumption and reaction rates [195].

Finally, hydrothermal and supercritical water treatment processes have been identified as innovative and minimally damaging remediation techniques [202,203]. Hydrothermal/subcritical water is defined as water at temperatures of 100 °C to 350 °C but sufficiently pressurized to remain a liquid, while supercritical water is defined as water at both temperatures and pressures higher than the critical point of water (374 °C, 22.1 MPa) [203]. High temperature and pressure create a highly reactive environment which is capable of catalyzing chemical reactions that cannot proceed at lower temperatures [202,203,204]. Water at these pressures and temperatures is able to reductively decompose PFAS, although the precise mechanism is not well understood [205,206]. Enhanced degradation has been observed with the addition of iron and the use of supercritical water over subcritical water [207]. However, the harsh environment created can cause corrosion, and heavy metals may leach into the water during treatment [202].

## 8. Emerging Research

### 8.1. Factors Affecting Remediation

Currently available commercial analytical techniques are limited to a small list of PFAS. They are unable to identify all PFAS compounds and treatment byproducts. However, there are advanced analytical techniques that are currently being developed (e.g., total oxidizable precursor (TOP) assays, particle-induced gamma-ray emission (PIGE) spectroscopy, adsorbable organic fluorine (AOF) paired with combustion ion chromatography (CIC), and high-resolution mass spectrometry techniques such as quadrupole time-of-flight (qTOF) to characterize unknown PFAS (e.g., precursors, polyfluorinated compounds). Those emerging analytical methods still remain at the research and development stage and are not commercially available [208,209,210,211].

A wide variety of remediation technologies that sequester or destroy PFAS have shown promise at the laboratory bench test- or field pilot-level, yet few have been field-demonstrated and are generally accepted. The current state of remediation technologies primarily focuses on treating PFOS and PFOA, the major PFAS compounds detected in drinking water and groundwater. However, many other PFAS may be present in environmental matrices including soil, wastewater, and biosolids. As mentioned, a diverse family of more than 4700 PFAS-related compounds with Chemical Abstract Service (CAS) Registry Numbers has been identified [5]. Uncertainties and a lack of knowledge about their properties, pharmacokinetics and toxicity, environmental fate, and human health risks remain a major obstacle and growing as the number of identified PFAS increase in quantity and complexity [14]. In particular, this concern involves the multiple new short-chain variant alternatives and new formulations (emerging PFAS, e.g., GenX) brought to the market. Remediation technologies that exploit the physiochemical properties of chemical compounds to transform, immobilize, or remove contaminants are usually generally successful. Remediation technologies that have been tested and are shown to be effective for only a narrow list of common PFAS compounds, but the effectiveness of those treatment technologies on other PFAS is unknown. Therefore, a more thorough understanding of the chemical structures and properties of PFAS is necessary for the improvement of remediation efficacy. Decreasing this knowledge and information gap is particularly important for understanding transformation pathways that may result in the formation of poorly understood intermediate or final fluorinated products that may present new risks.

Co-contaminants, including hydrocarbons and chlorinated solvents, are usually present alongside PFAS in contaminated media. Variations in geochemical constituents can further complicate the situation (e.g., temperature, pH, organic matter content, inorganic ions, oxygen concentrations, groundwater, sediment geochemistry). Hence, comprehensive research studies are needed to address the effects of complex field conditions on the analytical methods as well as the performance of treatment technologies.

### 8.2. New Perspectives on Treatment

The limited treatment technologies available for PFAS removal are currently restricted to drinking water and groundwater treatment. For other environmental media (e.g., AFFF, highly contaminated soil, and biosolids), cost-prohibitive and environmentally damaging high temperature incineration is currently the only proven and viable option. The feasibility of removing PFAS in these media has not been widely evaluated but warrants further research. A combination of multiple approaches that leverages both sequestration technologies and destruction technologies in a treatment train can maximize treatment efficiency, energy usage, cost, and sustainability. For example, ion-exchange can be combined with electrochemical, plasma, sonolysis, or hydrothermal processes. A comprehensive assessment of feasibility, performance, and cost of treatment is necessary for a given site to select the proper PFAS treatment technology based on treatment media, site characteristics and economics. Further research on the economic viability and treatment performance of emerging destructive technologies in combination with sequestration options will be needed if the cost and effectiveness of PFAS remediation are to be improved.

## 9. Summary and Conclusions

The growing awareness of the effects of PFAS on human and environmental health have led to researchers and the industry to identify them as toxic and bio-accumulative pollutants. PFAS have seen commercial use for less than a century, but the body of research on its sources, properties, fate and transport, and health effects have shown that it is surprisingly complex. Applied research on remediation and removal methods have proceeded in parallel with research on its basic science and health effects. Remediation has been constantly complicated by new information, and this manuscript seeks to provide a baseline understanding on these issues to encourage the development of novel treatment technologies.

Short-chain emerging PFAS have begun to replace long-chain legacy PFAS, but their environmental detection and negative health effects have continued to be noted. Regulations primarily concern PFOA and PFOS, the two most common legacy PFAS. At the same time, equal regulatory scrutiny deserves to be paid towards short-chain PFAS and their precursors. The current priority of researchers and policymakers alike should be to create uniform and actionable standards for protecting drinking water sources, while also creating opportunities for further research to better understand PFAS. A more thorough understanding of the behavior and nature of PFAS across this broad family will be conducive to the development of minimally damaging, efficient, and cost-effective remediation strategies.

Common field-scale treatments involve the use of adsorbents to concentrate and sequester PFAS, but these actions require the downstream treatment of highly concentrated secondary waste streams. Most of the available treatment technologies claim to treat PFOS and PFOA and some other legacy PFAS, but have not been assessed to treat the entire PFAS family, including emerging PFAS compounds. Furthermore, cost, environmental safety, size, time are competing concerns, with some existing technologies showing advantages over others in individual areas.

## Figures and Tables

**Table 1:** Physical and chemical properties of selected PFAS, adapted from ITRC PFAS Team [51].

Molecular Formula	Molecular Weight	Vapor Pressure at 25 °C (Pa)	Water Solubility at 25 °C (mg/L)	Melting Point (°C)	Boiling Point (°C)	Log of Organic Carbon Partition Coefficient (logK_oc_)
C_3_F_7_COOH	214.0	851	562,877	−17.5	120	1.9
C_4_F_9_SO_3_H	300.1	631	30,010	20.4–70.4	205–214	1.2–1.79
C_5_F_11_COOH	314.1	120	21,730	14	157	1.3
C_6_F_13_SO_3_H	400.1	58.9	2302	26.7–73.2	218–238	2.4–3.1
C_7_F_15_COOH	414.1	4.2	771	54.3	188	1.89–2.63
C_8_F_17_SO_3_H	500.1	6.8	60.1	15.2–73.5	219–262	2.4–3.7
C_9_F_19_COOH	514.1	0.2	25.2	78–88	218	2.76–2.96
C_10_F_21_SO_3_H	600.1	0.7	2.4	11.6–85.2	224–284	3.53–3.66
